# A descriptive follow-up interview study assessing patient-centred outcomes: Salford Lung Study in Asthma (SLS Asthma)

**DOI:** 10.1038/s41533-019-0142-x

**Published:** 2019-08-15

**Authors:** Lynda Doward, Henrik Svedsater, Diane Whalley, Rebecca Crawford, David Leather, James Lay-Flurrie, Nick Bosanquet

**Affiliations:** 1 RTI Health Solutions, Manchester, UK; 2 GlaxoSmithKline plc., Brentford, UK; 3 GlaxoSmithKline plc., Uxbridge, UK; 4 Imperial College London, London, UK

**Keywords:** Asthma, Inflammatory diseases

## Abstract

The Salford Lung Study in Asthma (SLS Asthma) was a multicentre, randomised, controlled, open-label trial that assessed initiating once-daily, single-inhaler fluticasone furoate/vilanterol (FF/VI) 100 μg/25 μg or 200 μg/25 μg versus continuing usual care. A subgroup (*n* = 400) from SLS Asthma was enrolled in this exploratory, interview-based follow-up study. Quantitative and qualitative data were collected via questionnaires. The primary objective was to capture patient-centred outcomes (symptom experience, quality of life [QoL], disease management behaviours) and patient experience. Secondary objectives were to assess the correlation of patient-reported outcomes with pre-defined variables from SLS Asthma (Asthma Control Test [ACT] score). The follow-up sample was representative of the SLS Asthma population; half reported asthma improvement during the study. Breathlessness was the most likely symptom to improve (47.8% of patients reported improvement). Most patients reported ‘no change’ in overall QoL (57.5%) and daily life domains (functioning 66.3%, activities 68.3%, relationships 86.8%, psychological 68.5%). Functioning was reported as the most frequently improved domain (29.8% of patients). Perceived improvement in asthma control (42.5%) and confidence (37.3%) was frequent. ACT responders (defined as patients achieving an ACT score ≥20 and/or an increase of ≥3 in ACT score from baseline at Week 52) were more likely to report asthma improvement (88.7% of patients reporting ‘a lot’ of improvement) than non-responders. Patients’ asthma experiences generally improved during SLS Asthma. Clinical improvements were often associated with perceived improvement by patients, particularly among ACT responders.

## Introduction

Asthma can have a profound impact on health-related quality of life (HRQoL), including disruption of daily activities, reduced physical functioning and sleep disturbance.^1–3^ Patients with asthma also report poor life satisfaction and health status, high psychological distress and anxiety associated with their condition.^4,5^

Historically, clinical trials in asthma have focussed largely on symptom minimisation and lung function as indicators of treatment outcome. There is, however, increasing evidence that these clinical indicators do not fully capture patients’ experiences of their disease. For example, patients may experience a change in health status that is not reflected by clinical assessments.^6^ As such, there is growing recognition of the importance of assessing HRQoL using validated patient-reported outcome questionnaires and qualitative interviews to capture patient experiences and preferences in a structured manner.^6,7^

The primary objective of asthma treatment is asthma control, which is defined by the extent of disease impact and future exacerbation risk.^8^ Asthma control is achieved using pharmacological approaches and, where relevant, non-pharmacological interventions, such as smoking cessation, education on healthy lifestyles and inhaler technique, breathing exercises, the use of personalised asthma management plans and psychological assessments.^8^ The importance of asthma control in disease management and its relationship with patient progress is recognised by the Global Initiative for Asthma, and poor asthma control is associated with reduced HRQoL.^9,10^ Thus, it is critical that researchers explore factors affecting the level of asthma control achieved in different patients in order to guide therapeutic intervention. To this end, the Asthma Control Test (ACT) was developed. The ACT is a validated, patient-based questionnaire for assessing asthma control,^11^ where scores ≤19 indicate not well-controlled asthma,^12^ although it does not capture information about HRQoL.

The Salford Lung Study in Asthma (SLS Asthma) was a 12-month, multicentre, randomised, controlled, open-label trial conducted in United Kingdom primary care, which assessed the effectiveness and safety of initiating once-daily, single-inhaler fluticasone furoate/vilanterol (FF/VI) 100 μg/25 μg or 200 μg/25 μg versus continuing usual care (UC) in patients with asthma.^13^ SLS Asthma showed that initiating FF/VI significantly improved asthma control (patients with a baseline ACT score <20 achieving an ACT score ≥20 and/or an increase of ≥3 in ACT score from baseline, defined as ‘ACT response’) without increasing the risk of serious adverse events when compared with continuing UC.

This exploratory, descriptive, follow-up interview study aimed to complement the findings from SLS Asthma by providing additional information on patient perceptions and experiences of treatment outcomes, collected through follow-up interviews conducted with a subset of patients who completed SLS Asthma.

## Results

### Representativeness of the SLS Asthma population

The follow-up sample (*n* = 400; all patients completing follow-up interviews) was found to be representative of the overall sample of patients who completed SLS Asthma (*N* = 3866) in terms of demographic and disease characteristics (Table 1). The follow-up sample was comparable to the overall population completing SLS Asthma in terms of mean (standard deviation [SD]) age at entry to SLS Asthma (48.4 [16.3] years versus 50.5 [16.2] years), gender (female 56.3 versus 58.9%) and ACT score at SLS Asthma baseline (mean [SD] 16.5 [4.5] versus 16.4 [4.4]). Similarity was also apparent in ACT score change from baseline and the percentage of patients randomised to initiation of FF/VI. Further sociodemographic, disease and lifestyle characteristics for the follow-up population are shown in Supplementary Table 1.

**Table 1:** Characteristics of the patients who completed SLS Asthma and who participated in the current follow-up study

	Total SLS Asthma completion sample (*n* = 3866)	SLS Asthma sample participating in follow-up interviews (*n* = 400)	Standard interview sample (*n* = 360)	Extended interview sample (*n* = 40)
Age at SLS Asthma baseline, years				
Mean (SD)	50.5 (16.2)	48.4 (16.3)	48.6 (16.5)	47.4 (14.4)
Median(range)	51.0 (18–91)	49.0 (18–86)	49.0 (18–86)	47.0 (21–76)
Gender, *n* (%)^a^				
Male	1589 (41.1)	175 (43.8)	156 (43.3)	19 (47.5)
Female	2277 (58.9)	225 (56.3)	204 (56.7)	21 (52.5)
ACT score at SLS Asthma baseline				
Mean (SD)	16.4 (4.4)	16.5 (4.5)	16.4 (4.5)	17.1 (5.0)
Median (range)	17.0 (5–25)	17.0 (6–25)	17.0 (6–25)	18.5 (7–25)
ACT score at SLS Asthma Week 52				
Mean (SD)	18.6 (4.8)	18.8 (4.7)	18.9 (4.7)	18.2 (4.9)
Median (range)	20.0 (5–25)	20.0 (7–25)	20.0 (7–25)	18.5 (8–25)
ACT score change between SLS Asthma baseline and Week 52				
Mean (SD)	2.1 (4.7)	2.3 (4.9)	2.4 (4.9)	1.1 (4.5)
Median (range)	2.0 (−17 to 19)	2.0 (−12 to 17)	2.0 (−12 to 17)	1.5 (−10 to 11)
SLS Asthma randomised treatment group, *n* (%)				
FF/VI	1920 (49.7)	199 (49.8)	177 (49.2)	22 (55.0)
UC	1946 (50.3)	201 (50.3)	183 (50.8)	18 (45.0)

*ACT* Asthma Control Test, *FF/VI* fluticasone furoate/vilanterol, *SD* standard deviation, *SLS Asthma* Salford Lung Study in Asthma, *UC* usual care

^a^One patient was listed as female in SLS Asthma and male in the follow-up; the reason for this change is unknown

Of the 400 patients in the follow-up sample, 360 completed standard interviews and 40 completed extended interviews. Demographic and clinical characteristics were similar for patients included in the standard interview and extended interview samples; however, marginally more patients in the extended interview sample had been randomised to initiate FF/VI than continue UC (49.2% of the standard interview sample versus 55.0% of the extended interview sample). Such variation is to be expected given the small sample size of the extended interview group.

### Overall asthma experience

Only 24 patients (6.0% of the follow-up sample) reported that their asthma got worse during SLS Asthma (4.3% got ‘a little’ worse; 1.8% got ‘a lot’ worse). A total of 176 patients (44.0%) reported that their asthma had not changed. The remaining 200 patients (50.0%) reported an overall improvement in their asthma since starting SLS Asthma (23.5% improved ‘a little’; 26.5% improved ‘a lot’).

The perceived severity of asthma symptoms over the 7 days prior to the follow-up interview was rated as ‘mild’ by 23.5% of the sample, ‘moderate’ by 18.3% and ‘severe’ by 2.3%. Just over half (56.0%) of the sample reported having no symptoms during this period.

### Asthma symptom experience

**Fig. 1:**
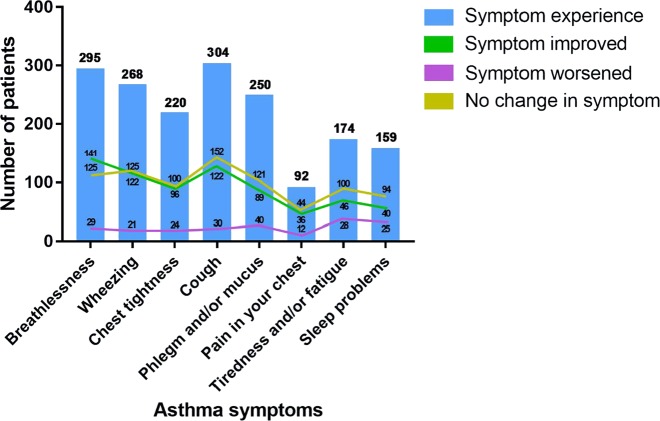
Asthma symptom experience during SLS Asthma

In the overall follow-up sample, the most frequently reported symptoms experienced during SLS Asthma were cough (76.0%), breathlessness (73.8%) and wheezing (67.0%), summarised in Table 2. Chest tightness and phlegm and/or mucus were also reported by more than half of the follow-up sample (55.0% and 62.5%, respectively; Table 2). Figure 1 shows the number of patients reporting each symptom to have improved, stayed the same or worsened since the start of SLS Asthma. More patients reported improvement than reported worsening for each symptom.

**Table 2:** Asthma symptoms experienced during SLS Asthma, self-management strategies used by participants for their asthma and avoidance of environmental trigger factors

	SLS Asthma sample participating in follow-up interviews (*n* = 400)
Symptoms, *n* (%)	
Breathlessness	295 (73.8)
Wheezing	268 (67.0)
Chest tightness	220 (55.0)
Cough	304 (76.0)
Phlegm and/or mucus	250 (62.5)
Pain in your chest	92 (23.0)
Tiredness and/or fatigue	174 (43.5)
Sleep problems	159 (39.8)
Extent to which participants reported using each strategy to help self-manage their asthma, *n* (%)	
Pace yourself or do things more slowly	
Very much/quite a lot/a little	16 (4.0)/63 (15.8)/130 (32.5)
Not at all	191 (47.8)
Plan activities carefully	
Very much/quite a lot/a little	15 (3.8)/62 (15.5)/67 (16.8)
Not at all	256 (64.0)
Accept help for everyday tasks	
Very much/quite a lot/a little	13 (3.3)/24 (6.0)/71 (17.8)
Not at all	292 (73.0)
Use rescue inhaler before exercise^a^	
Very much/quite a lot/a little	42 (10.5)/60 (15.0)/112 (28.0)
Not at all	182 (45.5)
Keep inhalers close at hand	
Very much/quite a lot/a little	208 (52.0)/105 (26.3)/39 (9.8)
Not at all	48 (12.0)
Take regular exercise^b^	
Very much/quite a lot/a little	47 (11.8)/132 (33.0)/101 (25.3)
Not at all	112 (28.0)
Avoid triggers	
Very much/quite a lot/a little	37 (9.3)/128 (32.0)/116 (29.0)
Not at all	119 (29.8)
Patients avoiding trigger, *n* (%)	
Places with air conditioning or central heating	63 (15.8)
Being exposed to people with coughs and colds	159 (39.8)
Places where there is smoke, dust or fumes	264 (66.0)
Exercise or physical exertion	127 (31.8)
Places where there are pets or animals	98 (24.5)

*SLS Asthma* Salford Lung Study in Asthma

^a^Missing *n* = 4 (1.0%)

^b^Missing *n* = 8 (2.0%)

Breathlessness was the symptom most likely to be reported as having improved since the start of SLS Asthma (47.8% of patients), followed by wheezing (45.5%) and chest tightening (43.7%). Of the 34 patients in the extended interview sample who reported breathlessness and/or shortness of breath and wheezing during SLS Asthma, 18 (52.9%) noted that, through affecting activities such as walking and climbing stairs, this symptom had the biggest impact on their daily life.

Cough was experienced by 87.5% of the extended interview sample, but only seven patients (17.5%) reported cough as the symptom with the greatest impact on their daily life. Of these, three patients also commented on the association between cough and phlegm/mucus. One female patient (aged 54 years) mentioned embarrassment regarding mucus in social situations and one male patient (aged 45 years) commented on the negative effect that phlegm/mucus had on his relationship.

Chest tightness was reported as the symptom with the largest day-to-day impact by five patients in the extended interview sample. One patient (female, aged 70 years) reported that she was unable to distinguish between her asthma and angina, saying: “It’s the tightness of the chest. I don’t like that, it frightens me.” Breathing/breathlessness was the asthma symptom that patients in the extended interview sample most commonly said they would like to improve the most (10/40 patients, 25%).

### Impact of asthma on daily life and QoL

**Fig. 2:**
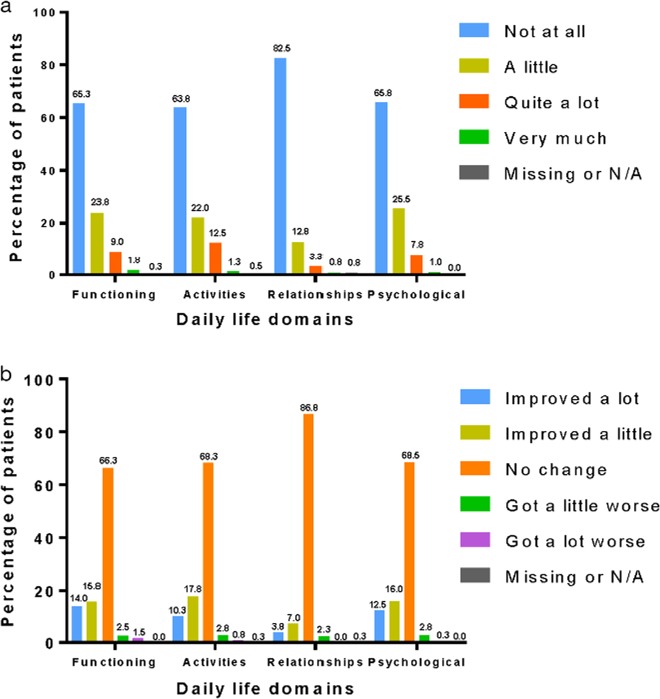
**a** Effect of asthma on quality of life (QoL) per daily life domains. **b** Perceived change in the impact of asthma on QoL per daily life domain

The interview schedule included questions on the impact of asthma on different activities/issues within the four domains of daily life (functioning, activities, relationships and psychological well-being), and responses were scored on a 4-point scale, ranging from ‘none at all’ (score 1) to ‘very much’ or ‘unable to do’ (score 4). For the purpose of the analysis, ‘very much’ and ‘unable to do’ were classed as a single category. For each of these four domains, the majority of patients reported that asthma had little or no effect (Fig. 2a).

The domain least impacted by asthma was relationships; 82.5% of patients in the follow-up sample responded that asthma did not affect their relationship. Similarly, over half of the participants in the extended interview sample reported that their asthma had no impact on social or family events. However, willingness to participate in an event or outing was often determined by the anticipated level of activity associated with the event or the anticipated presence of known asthma triggers. Some participants reported that their desire to avoid experiencing breathlessness on exertion, or to avoid a known asthma trigger, had led them to miss out on excursions with family and friends.

Changes in daily life domains since the start of SLS Asthma were scored on a 5-point scale, ranging from ‘improved a lot’ (score 1) to ‘got a lot worse’ (score 5). The majority of patients reported ‘no change’ in all four domains (functioning 66.3%; activities 68.3%; relationships 86.8%; psychological well-being 68.5%). More patients reported improvements for each domain than reported worsening, and the domain perceived to be the most improved was functioning (14.0% improved ‘a lot’; 15.8% improved ‘a little’; Fig. 2b).

The mean overall QoL in the follow-up sample, as scored on a 10-point scale ranging from worst possible QoL (score 1) to best possible QoL (score 10), was 7.8 (SD 1.7; range 2.0–10.0). For perceived change in overall QoL, assessed using the same 5-point scale that was used for daily life domains, 57.5% of patients reported ‘no change’ in their overall QoL since the start of SLS Asthma. Approximately a third (36.3%) reported that, since the start of SLS Asthma, their overall QoL had improved by either ‘a little’ (19.0%) or by ‘a lot’ (17.3%). Only 6.3% reported that their QoL had worsened. The mean change in QoL score was not validated for clinical significance in the follow-up sample.

### Asthma triggers and self-management

The most commonly reported asthma triggers among patients in the overall follow-up sample were places with smoke, dust or fumes (82.8% of patients) and exercise or physical exertion (68.8%); 66.0% and 31.8% of patients, respectively, reported that they actively avoid these triggers. Most patients reported that the impact of environmental triggers on asthma symptoms was unchanged since the start of SLS Asthma. Nonetheless, exercise or physical exertion was the trigger reported most likely to have less effect since the start of SLS Asthma. Patients in the extended interview sample also reported that they avoided triggers to prevent the worsening of symptoms but acknowledged that avoidance is difficult and, at times, not possible.

Of the seven self-management strategies presented in the interview schedule, ‘keep inhalers close at hand’ was the most commonly employed in the follow-up interview sample, with a median response of ‘very much’ (Table 2). Commonly reported strategies for disease management also included ‘take regular exercise’, ‘avoid triggers’, ‘use rescue inhaler before exercise’ and ‘pace yourself or do things more slowly’ (median response ‘a little’; Table 2). Other strategies were also proposed by patients in the extended interviews, such as nose-breathing, and taking antihistamines prior to exposure to factors that were likely to exacerbate their asthma.

### Feelings of control and confidence

At the time of the follow-up interview, the majority of patients in the follow-up sample reported that they had control over their asthma (46.5% ‘quite a lot’; 36.0% ‘very much’) and were confident in their ability to manage their asthma (40.3% ‘quite a lot’; 45.8% ‘very much’). Of the 17 patients in the extended interview sample reporting that they were ‘very much’ in control of their asthma, one male (aged 43 years) attributed this to frequent inhaler use and improved adherence, saying: “It’s improved because I normally didn’t take anything every day, but now sometimes I do miss when I’m rushing for work and miss it, but I’m getting used to taking that day by day.”

**Fig. 3:**
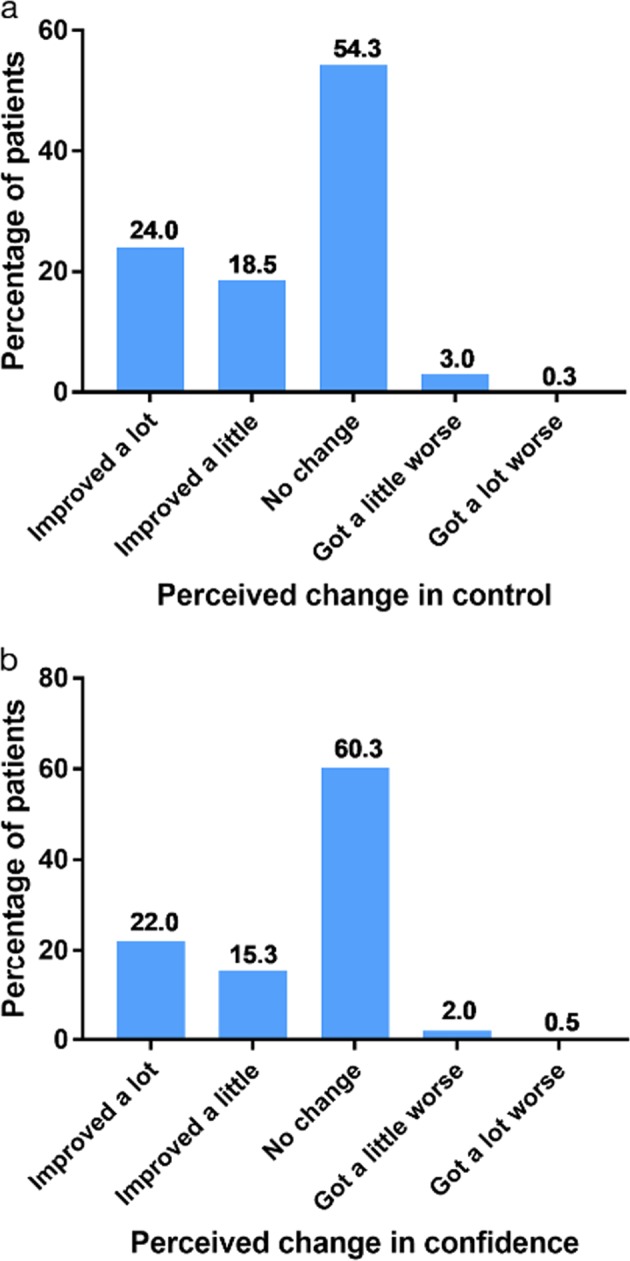
**a** Perceived change in asthma control since the start of SLS Asthma. **b** Perceived change in confidence since the start of SLS Asthma

In the follow-up sample, most patients reported no change or an improvement (‘a little’ or ‘a lot’) in both their perceived asthma control (Fig. 3a) and their confidence to control their asthma (Fig. 3b). ‘A lot’ of improvement, ‘a little’ improvement and ‘no change’ were reported by 24.0, 18.5 and 54.3% of patients, respectively, for asthma control. For confidence, the respective percentages were 22.0, 15.3 and 60.3%. Very few patients reported a worsening in control (3.3%) or confidence (2.5%) since the start of SLS Asthma.

### Experience and management of asthma attacks

Almost one-quarter (24.0%) of the follow-up sample reported that they kept a supply of emergency prescription medication to treat an asthma attack, and more than half (59.0%) reported that they had experienced an asthma attack in the past. Of these patients, the perceived severity of attack was reported by patients as mild (14.0%), moderate (44.5%), severe (31.8%) or very severe (9.7%). The most commonly reported management strategies for short-term relief of symptoms were ‘take more puffs of your usual rescue inhaler’ (81.8%) and ‘rest up completely’ (55.9%). In terms of additional medications for their last attack, 33.1% of patients had taken oral steroids, 25.4% had used a nebuliser and 19.9% had taken antibiotics.

Many patients sought medical help for their last attack (55.1% of the 236 patients who reported ever having an asthma attack), largely through an emergency service (46.9%) or from their general practice (42.3%). Patients in the extended interview sample provided further detail on the decision-making process for managing an attack, with the severity of symptoms described as a key influence. The time of day at which the attack occurred also factored into some patients’ decision-making process, with a night-time attack increasing the likelihood of seeking immediate, emergency medical help, particularly if they saw no improvement in their symptoms over a period of time.

### Association between ACT response and patient-centred outcomes

**Fig. 4:**
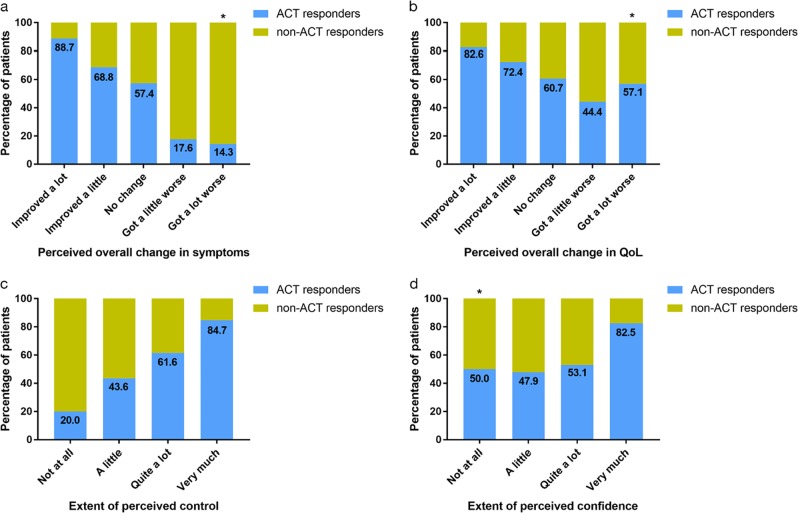
Percentage of Asthma Control Test (ACT) responders and ACT non-responders in each response category for **a** perceived overall change in asthma since the start of SLS Asthma, **b** perceived overall change in QoL since the start of SLS Asthma, **c** perceived disease control and **d** perceived confidence

Analyses by ACT response were conducted with 399 patients; Week 52 ACT scores were not available for 1 patient (Fig. 4a). Of the patients who reported ‘a little’ or ‘a lot’ of overall improvement in their asthma since the start of SLS Asthma, 68.8% and 88.7%, respectively, were ACT responders. In terms of specific symptoms, >60% of patients who reported ‘a lot’ of improvement in breathlessness, wheezing, chest tightness, cough, phlegm/mucus, chest pain, tiredness and sleep problems were ACT responders. Few patients reported that their overall asthma or their specific symptoms worsened ‘a lot’ since the start of SLS Asthma (*n* < 10); no responders reported that the symptoms of breathlessness, wheezing and chest pain got ‘a lot’ worse.

Across the four daily life domains (functioning, activities, relationships, psychological), there was a trend for higher percentages of ACT responders among patients who reported the life domain to have improved (range: 77.5–87.8%) than among patients whose symptoms had not changed (range: 61.5–65.6%) or had worsened (range: 0.0–66.7%) (Fig. 4b).

Patients who perceived an improvement in their disease control and confidence to control their asthma were also more likely to be ACT responders than non-responders (Fig. 4c, d). Of the patients reporting ‘very much’ control and confidence, 84.7% and 82.5%, respectively, were ACT responders. As the extent of control that patients reported over their asthma decreased (from ‘very much’ to ‘quite a lot’, ‘a little’ and ‘not at all’), the proportion of patients who were ACT responders also decreased. For confidence, this trend was almost mirrored, but 50.0% of patients who reported that they were ‘not at all’ confident in controlling their asthma and 47.9% of patients who reported ‘a little’ confidence were ACT responders.

## Discussion

We present the findings of an exploratory, descriptive, follow-up study to complement the overall results from SLS Asthma and to capture patient-centred information beyond the scope of SLS Asthma. The follow-up sample was deemed to be representative of patients who completed SLS Asthma in terms of gender, ACT scores and the proportion of patients in each treatment group. Mean age differed slightly between these populations due to the 1-year SLS Asthma study duration. The results obtained in this follow-up study largely complemented the results of the primary SLS Asthma analysis and thus may be considered applicable to patient experiences in real-world asthma treatment.

This study involved a focus on patient experience, including the patient’s perception of their asthma symptoms and QoL. Such patient-focussed outcomes are becoming increasingly popular in contemporary clinical studies due to their ability to capture patients’ contributions to, and perception of, their own healthcare.^14^

Half of patients in the follow-up sample reported that their asthma had improved ‘a little’ or ‘a lot’ since the start of SLS Asthma. Supporting this, a previous large-scale survey of European patients found that a large proportion of individuals who had experienced an acute asthma exacerbation in the prior year considered their asthma to be controlled.^15^ In addition, we found that ACT responders (patients achieving an ACT score ≥20 and/or an increase of ≥3 in ACT score from baseline at Week 52) were more likely than ACT non-responders to report improvement in their asthma symptoms and overall asthma at the follow-up interview; this may be indicative of greater engagement in asthma management among ACT responders compared with ACT non-responders.

Both the quantitative and qualitative aspects of this study identified that asthma symptoms such as cough, breathlessness and chest tightness were common among patients and had the potential to impact central domains of patient QoL. Previous studies have also highlighted that these common symptoms, particularly cough and breathlessness, and their impact on ability to carry out activities are considered to be the aspects of patients’ asthma that they dislike the most.^16,17^ In the qualitative portion of this study, these symptoms and their worsening were also found to be associated with changes in lifestyle to limit their effect. In the overall follow-up sample, 68.8% of participants reported that exercise or physical exertion was a trigger for their asthma symptoms, with 32.0% of participants reporting that they generally tried to avoid all asthma triggers and 31.8% avoiding exercise specifically. Although avoidance of physical activity due to exacerbation of asthma symptoms is a common phenomenon among asthma patients, it may lead to long-term health risks,^18,19^ such as obesity and psychological problems, which negatively impact patient wellbeing.^20,21^ Subsequently, interventions to encourage positive lifestyle changes and exercise among asthma patients become important in minimising long-term health risks.^19^ Indeed, when considering potential asthma management strategies in this study, although 28.0% of participants responded ‘not at all’ to ‘taking regular exercise’, the remainder responded positively, thereby suggesting that most respondents were aware that regular exercise is beneficial for asthma management. Nonetheless, approximately half of participants were obliged to ‘pace yourself or do things more slowly’, and, in the extended interviews, some participants reported continuing with activities regardless of their asthma symptoms but were reliant on their inhalers, while others recognised that exercise would improve their general health as well as their asthma. These study observations emphasise the complexity of asthma management and the need for in-depth patient assessments in conjunction with step-wise changes to treatment when aiming to achieve optimal asthma control.^8^

In addition to patients listing symptoms that they disliked most, many patients in this follow-up study appeared unable to fully differentiate between their asthma symptoms when describing which had the biggest impact on their day-to-day lives. In particular, wheezing, breathlessness and chest tightness were sometimes used interchangeably by participants when describing their symptoms. This could be a reflection of a lack of education about asthma and its symptoms and is perhaps indicative of a greater need for patient/physician interaction when assessing the most effective asthma treatment. For instance, previous studies have identified discordance between patient and physician knowledge with relation to asthma severity and perceptions of asthma control.^16,22,23^ Improved patient/physician interactions could thus be a very important tool for improving patient understanding, communication and management of their own disease and is an area that could benefit from further studies conducted in healthcare practices.

The majority of participants reported that asthma had no impact on the four main QoL domains of daily life (relationships [82.5%]; psychological well-being [65.8%]; functioning [65.3%]; and activity [63.8%]), and similar proportions reported no change in these domains during SLS Asthma. This was surprising as prospectively collected patient-reported outcomes data, based on results from a validated instrument, have previously found a HRQoL benefit of initiating FF/VI treatment during SLS Asthma, compared with continuing UC.^17^ For those participants who did report an impact of asthma, functioning, activities and psychological impacts were reported more commonly than impacts on relationships; changes reported during SLS Asthma in all four domains largely represented improvements. Social life was reported to be affected by their condition in some patients. The responses received in extended interviews indicated that this is largely due to a desire to avoid experiencing breathlessness on exertion, or to avoid a known asthma trigger, which led some patients to miss excursions with family and friends.

A substantial proportion of patients in the follow-up sample reported good control of their asthma and confidence to control their asthma. More patients also reported these parameters to have improved since the start of SLS Asthma than the number who reported it to have worsened, although most patients reported no change. Interestingly, the majority of patients who reported that they were ‘very much’ confident regarding their asthma control and confidence were ACT responders in SLS Asthma (84.7% and 82.5%, respectively). Nevertheless, of the patients reporting that they were ‘not at all’ confident in their ability to control their asthma, 50.0% were ACT responders. In the qualitative portion of the study, ‘keep inhalers close at hand’ was shown to be by far the most popular self-management strategy among patients in the extended interview sample. Approximately one quarter of participants reported using antibiotics to treat their last attack. While this behaviour may reflect inappropriate antibiotic prescribing, it is also possible that their asthma exacerbations may have been associated with a chest infection. Overall, evidence for effectiveness of antibiotics in reducing asthma exacerbations is inconclusive and must be balanced against the risk of antibiotic resistance;^24^ however, treatment with macrolide-type antibiotics has been shown to reduce exacerbations in patients with persistent asthma that remains uncontrolled despite medium-to-high dose inhaled corticosteroid/long-acting beta-agonist therapy.^25^

As this follow-up study used the SLS Asthma patient pool, the data gathered are strengthened by their potential to be explored in relation to the original clinical outcomes. As such, the data presented here, both quantitative and qualitative in nature, effectively expand on those obtained in SLS Asthma and facilitate further insight into the patient experience during this large clinical trial. Furthermore, the collection of qualitative data on patient-reported outcomes facilitates a focus on the experiences of individuals, which is seldom captured in the context of a clinical study alone.

This study was limited primarily by its exploratory nature; this allows the consideration of a broad range of issues of potential relevance to outcomes in asthma but limits the inferences that can be made from the results and precludes definitive conclusions being drawn. In addition, caution should be taken when generalising results to the wider asthma population. Any findings need to be verified in future studies designed to test specific hypotheses. Furthermore, as the study relied on the patient’s recollection of events occurring throughout the 1-year study period of SLS Asthma, results may be prone to an extent of recall bias. Patient uncertainty in distinguishing between their symptoms may have also confounded how these symptoms were reported. The open-label nature of SLS Asthma, specifically the un-validated endpoints/questions posed in the follow-up interviews, may have influenced participants’ perceptions of the treatments and so comparisons between them have not been included in the manuscript. The lack of statistical analysis conducted during the follow-up study may limit our interpretation of the true extent of the difference between parameters. Statistical analysis would be inappropriate, however, due to the involvement of a potentially self-selecting population of patients who were randomised at an earlier time point.

## Conclusion

Results from interviews on outcomes, including change in overall asthma, symptom experience and QoL per central QoL domains, showed improvement throughout SLS Asthma in our follow-up population. Improvement was also more commonly reported by ACT responders from SLS Asthma than non-responders. Furthermore, we were able to capture the specific ways in which patients perceived these improvements, and worsening where applicable, through the qualitative portion of the study. Initiation of FF/VI was associated with more frequently perceived asthma improvement than continuing UC. In all, our study provides valuable insight into the patient’s perception and experience of their asthma treatment that may inform healthcare decision-making in the future.

## Methods

### Study objectives

The primary objectives of this interview-based follow-up study were to describe: the background and lifestyle characteristics of patients taking part in SLS Asthma; patient-centred outcomes beyond those captured by standardised instruments administered in SLS Asthma, including symptom experience, sleep, impact on daily life and overall QoL; patients’ experiences, perceptions and management of disease, focusing on disease awareness, self-management strategies and treatment-seeking behaviours; patients’ attitudes towards, and potential barriers to, medication adherence.

The secondary objectives were to explore how selected patient characteristics and patient-centred outcomes relate to key variables in SLS Asthma, specifically asthma control response per ACT scores and randomised treatment group.

### Study design

This was an exploratory, qualitative study involving follow-up interviews in a subgroup of patients (*n* = 400) with asthma who had completed SLS Asthma.^13^ A mixed-methods approach was taken. Quantitative data were collected from all patients using study-specific, structured, closed-ended questions. These data were used to describe the characteristics, experiences and perceptions of patients in the SLS Asthma sample beyond the information captured in the main study. Additional qualitative data were collected from a subset of 40 patients selected at random from the overall follow-up sample, using semi-structured, open-ended questions on key topic areas. For patients who completed the closed-ended questions only, the interviews are termed ‘standard interviews’; for the subset of patients who completed both the closed- and open-ended questions, the interviews are termed ‘extended interviews’.

This follow-up interview study was approved by the Proportionate Review Sub-Committee of the Health Research Authority (formerly the National Research Ethics Service) East Midlands Research Ethics Committee in August 2013 and GlaxoSmithKline plc.’s Protocol Review Committee in August 2015.

### Patient recruitment

Patients who completed SLS Asthma (i.e. attended the end-of-study [EOS] visit) and who were able to provide written informed consent and participate in a qualitative interview were eligible for inclusion.

A modified consecutive sampling approach was used to identify patients for recruitment: the first 400 patients who completed SLS Asthma, who agreed to take part and who met the study criteria (able to provide informed consent and participate in a qualitative interview) were included in the study. The SLS community team nursing staff presented the study to SLS Asthma patients as they attended their EOS visit. The first 400 patients who met the study inclusion criteria were enrolled. We planned to recruit every tenth patient enrolled to the extended interview sample. Following completion of 327 follow-up interviews, including 27 extended interviews, every seventh patient enrolled was selected for extended interviews to ensure the quota of 40 patients was met.

### Follow-up interview procedure

Follow-up interviews were conducted within 2 weeks of the patient’s EOS visit. A follow-up interview schedule was developed based on a targeted literature review and concept elicitation interviews conducted with 20 patients with asthma.

The interview schedule comprised 83 closed-ended questions (i.e. questions used in both the standard and extended interviews) and 17 open-ended questions (i.e. questions used in the extended interviews only). The schedule included questions on demographics, symptoms, impact on daily life, trigger factors, self-management and disease awareness, experience and management of asthma attacks, treatments, QoL and background and lifestyle. Patients also completed two standardised patient-reported outcomes instruments (COPD and Asthma Sleep Impact Scale^26^ and Adherence Starts With Knowledge-12^27^ [data not reported]). Interviews were conducted via telephone or face-to-face by trained interviewers using the structured interview schedule. Patients’ responses were recorded using an electronic data capture application designed specifically for this study.

### Statistical analyses

Using Cochran’s formulae for determining sample size, it was estimated that a sample size of 350–400 patients would provide 95% confidence that a given outcome in the overall SLS Asthma population would be within a 4.7–5.0% margin of error of the outcome obtained in the follow-up sample. As the final sample was 400 patients of an initial 3866, the margin of error was calculated to be 4.6% for results obtained in the follow-up study versus the overall SLS Asthma population. A sample size of 40 for the extended interviews was considered sufficient to provide qualitative data on target topics of interest.

Quantitative analysis of the closed-ended question data was descriptive only, with no inferential statistical tests performed; the analysis was conducted using SAS 9.4 (SAS Institute; Cary, NC). Descriptive summary statistics were calculated for all analysis variables for the overall follow-up interview sample. A qualitative description approach was used for the analysis of the extended interview data;^28,29^ a coding framework was implemented to aid the analysis of the open-ended responses.

Secondary analyses were conducted to describe the association between selected interview variables and outcomes from the overall SLS Asthma data set (ACT response and randomised treatment). For the asthma control analysis, patients were categorised as ACT responders (patients achieving an ACT score ≥20 and/or an increase of ≥3 in ACT score from baseline at Week 52) or ACT non-responders (patients with a total ACT score <20 and a change from baseline in ACT score of <3 at Week 52). For the treatment group analysis, patients were categorised according to their randomised treatment group in SLS Asthma: initiated FF/VI versus continued UC.

### Reporting summary

Further information on research design is available in the Nature Research Reporting Summary linked to this article.

## Supplementary information

Supplemental material

Reporting Summary

## Data Availability

GlaxoSmithKline plc. makes available anonymised individual participant data and associated documents from interventional clinical studies that evaluate medicines upon approval of proposals submitted to www.clinicalstudydatarequest.com. To access data for other types of GlaxoSmithKline plc.-sponsored research, for study documents without patient-level data and for clinical studies not listed, please submit an enquiry via the website.

## Supplementary information

**Supplementary Information** accompanies the paper on the *npj Primary Care Respiratory Medicine* website (10.1038/s41533-019-0142-x).
