# Supplementary material for: A descriptive follow-up interview study assessing patient-centred outcomes: Salford Lung Study in Asthma (SLS Asthma)
Source: NPJ Prim Care Respir Med. 2019 Aug 15;29:31. doi: 10.1038/s41533-019-0142-x (PMC6695403; doi:10.1038/s41533-019-0142-x)
Supplement: Supplementary file 1 — Supplemental material [file 41533_2019_142_MOESM1_ESM.docx]

**SUPPLEMENTARY APPENDIX**

**Text summary:** Through our follow-up interviews, we were able to obtain a large volume of data on the backgrounds of participating patients, including sociodemographic, disease and lifestyle information. We have provided this information in our supplement (Supplementary Table 1) to provide additional context to the results presented in the main manuscript [49/50 words].

**Supplementary Table 1.** Sociodemographic information, disease characteristics and lifestyle factors of the overall SLS Asthma follow-up sample

|  | Overall SLS Asthma follow-up sample  (*N* = 400) |
| --- | --- |
| Age at the follow-up interview, years | |
| Mean (SD) | 49.4 (16.3) |
| Median (range) | 50.0 (19–86) |
| Relationship status, *n* (%) | |
| Married/living as married/civil partnership | 239 (59.8) |
| Divorced/separated | 28 (7.0) |
| Widowed/surviving partner | 15 (3.8) |
| Single | 111 (27.8) |
| Other | 7 (1.8) |
| Housing situation, *n* (%) | |
| Homeowner | 231 (57.8) |
| Tenant/lodger - private rented property | 88 (22.0) |
| Tenant/lodger - public rented property | 58 (14.5) |
| Living with parents/family^a^ | 22 (5.5) |
| Other | 1 (0.3) |
| Children under 18 years old living at home, *n* (%) | |
| Yes | 104 (26.0) |
| No | 220 (55.0) |
| Missing | 76 (19.0) |
| Children age^b^ | |
| Pre-school age (0–4 years) | 39 (37.5) |
| Primary school age (5–10 years) | 38 (36.5) |
| Secondary school age (11–17 years) | 58 (55.8) |
| Employment status, *n* (%) | |
| Working full time | 169 (42.3) |
| Working part time | 61 (15.3) |
| Long-term sick leave | 21 (5.3) |
| Retired | 86 (21.5) |
| Unemployed | 30 (7.5) |
| Homemaker | 14 (3.5) |
| Student | 11 (2.8) |
| Other | 8 (2.0) |
| Change in employment status since start of SLS Asthma, *n* (%) | |
| Yes | 25 (6.3) |
| No | 375 (93.8) |
| Are you self-employed?^c^ *n* (%) | |
| Yes | 39 (17.0) |
| No | 191 (83.0) |
| How active is your job?^c^ *n* (%) | |
| Active (e.g. involves physical labour) | 80 (34.8) |
| Sedentary (e.g. office/desk-based) | 84 (36.5) |
| Both active and sedentary | 66 (28.7) |
| Asthma interferes with day-to-day productivity or efficiency,^d^ *n* (%) | |
| Yes | 40 (15.7) |
| No | 213 (83.5) |
| Not relevant | 1 (0.4) |
| Missing | 1 (0.4) |
| Age at asthma diagnosis, years | |
| Mean (SD) | 26.7 (20.2) |
| Median (range) | 25.0 (0–81) |
| Duration of asthma, years | |
| Mean (SD) | 22.7 (15.0) |
| Median (range) | 21.0 (0–74) |
| Diagnosis in childhood or adulthood, *n* (%) | |
| Childhood | 165 (41.3) |
| Adulthood | 235 (58.8) |
| Other long-term illness/ health problems,^e^ *n* (%) | |
| Condition that limits physical activities or mobility | 65 (16.3) |
| Psychological or emotional condition | 32 (8.0) |
| Other | 88 (22.0) |
| None | 243 (60.8) |
| How busy is your life?^f^ *n* (%) | |
| Not at all | 76 (19.0) |
| A little | 82 (20.5) |
| Quite a lot | 172 (43.0) |
| Very much | 124 (31.0) |
| Mean (SD) | 3.0 (0.9) |
| Median (interquartile range) | 3.0 (2.0–4.0) |
| How stressful is your life?^f^ *n* (%) | |
| Not at all | 76 (18.0) |
| A little | 142 (35.5) |
| Quite a lot | 119 (29.8) |
| Very much | 63 (15.8) |
| Mean (SD) | 2.4 (1.0) |
| Median (interquartile range) | 2.0 (2.0–3.0) |
| Do you have enough time for rest and relaxation?^f^ *n* (%) | |
| Not at all | 97 (24.3) |
| A little | 107 (26.8) |
| Quite a lot | 132 (33.0) |
| Very much | 64 (16.0) |
| Mean (SD) | 2.4 (1.0) |
| Median (interquartile range) | 2.0 (2.0–3.0) |
| How active is your lifestyle? *n* (%) | |
| Mostly inactive | 17 (4.3) |
| Activity levels are low | 69 (17.3) |
| Activity levels are moderate | 226 (56.5) |
| Activity levels are high | 88 (22.0) |
| Dog ownership, *n* (%) | |
| Yes | 107 (26.8) |
| No | 292 (73.0) |
| Missing | 1 (0.3) |
| Dog walking,^g^ *n* (%) | |
| Every day or nearly every day | 57 (53.3) |
| Once or twice a week | 18 (16.8) |
| Less than once a week | 5 (4.7) |
| Never | 27 (25.2) |
| Current alcohol consumption per week, *n* (%) | |
| 0 units | 150 (37.5) |
| 1–7 units | 131 (32.8) |
| 8–14 units | 57 (14.3) |
| 15–21 units | 33 (8.3) |
| 22–35 units | 19 (4.8) |
| 36–50 units | 6 (1.5) |
| 51 or more units | 4 (1.0) |
| Stressful life during SLS Asthma, *n* (%) | |
| Yes | 177 (44.3) |
| No | 223 (55.8) |

*SD* standard deviation

*SLS Asthma* Salford Lung Study in Asthma

^a^Category based on free-text response associated with ‘other’ category

^b^Reported only for patients who responded that they have children under the age of 18 years currently living with them (*n* = 104); respondents could select more than one response

^c^Reported only for patients who responded that their employment status was working full time or part time (*n* = 230)

^d^Reported only for patients who responded that their employment status was working full time or part time, homemaker, or student (*n* = 255)

**^e^**Patients were allowed to select more than one response

^f^Variable scored as 1 = not at all, 2 = a little, 3 = quite a lot, 4 = very much

^g^Reported only for patients who responded yes to dog ownership (*n* = 107)
